# Retraction Note: Suppressor capacity of copper nanoparticles biosynthesized using *Crocus sativus* L. leaf aqueous extract on methadone-induced cell death in adrenal phaeochromocytoma (PC12) cell line

**DOI:** 10.1038/s41598-020-77741-4

**Published:** 2020-11-30

**Authors:** Peng Zhang, Jian Cui, Shirin Mansooridara, Atoosa Shahriyari Kalantari, Akram Zangeneh, Mohammad Mahdi Zangeneh, Nastaran Sadeghian, Parham Taslimi, Ramazan Bayat, Fatih Şen

**Affiliations:** 1 Department of Neurosurgery, Henan Provincial People’s Hospital, People’s Hospital of Zhengzhou University, Medical College of Henan University, Zhengzhou, 450003 Henan China; 2 Department of Neurosurgery, Xi’an No. 1 Hospital, No. 30 South Street Powder Lane, Beilin District, Xi’an, 710002 Shaanxi China; 3 Medical Sciences Research Center, Faculty of Medicine, Tehran Medical Sciences Branch, Islamic Azad University, Tehran, Iran; 4 Department of Neurology, Faculty of Medicine, Tehran Medical Sciences, Islamic Azad University, Tehran, Iran; 5 Department of Clinical Sciences, Faculty of Veterinary Medicine, Razi University, Kermanshah, Iran; 6 Biotechnology and Medicinal Plants Research Center, Ilam University of Medical Sciences, Ilam, Iran; 7 Department of Chemistry, Faculty of Science, Atatürk University, 25240 Erzurum, Turkey; 8 Department of Biotechnology, Faculty of Science, Bartin University, 74100 Bartin, Turkey; 9 Sen Research Group, Department of Biochemistry, University of Dumlupınar, 43000 Kütahya, Turkey

Retraction of: *Scientific Reports* 10.1038/s41598-020-68142-8, published online 15 July 2020

The Editors have retracted this article.

After publication it was brought to the Editors' attention that some of the figures in this article appear to contain images duplicated from other sources. Specifically:

- Figure 1 appears to be partly duplicated from Figure 4 in Yan et al. [1], which reports a different set of experiments;
- Figure 4 appears to be duplicated from Figure 3 in Yan et al. [1], which reports a different set of experiments.

The authors provided raw data for all figures containing graphs. However, the Editors were not able to replicate the error bars presented in Figures 7-12 from these data. In addition, some of the data reported in Figure 9 appear to be below the detection limit of the assay used to generate them.

The Editors therefore no longer have confidence in the integrity of the data presented.

Jian Cui, Mohammad Mahdi Zangeneh, Nastaran Sadeghian, Parham Taslimi, and Fatih Şen disagree with the retraction. Peng Zhang, Shirin Mansooridara, Atoosa Shahriyari Kalantari, Akram Zangeneh, and Ramazan Bayat did not respond to the correspondence about the retraction.

## References

[1] Yan W (2020). Chemical characterization and neuroprotective properties of copper nanoparticles green-synthesized by nigella sativa L. seed aqueous extract against methadone-induced cell death in adrenal phaeochromocytoma (PC12) cell line. J. Exp. Nanosci..

